# Landau levels and magneto-transport property of monolayer phosphorene

**DOI:** 10.1038/srep12295

**Published:** 2015-07-10

**Authors:** X. Y. Zhou, R. Zhang, J. P. Sun, Y. L. Zou, D. Zhang, W. K. Lou, F. Cheng, G. H. Zhou, F. Zhai, Kai Chang

**Affiliations:** 1 SKLSM, Institute of Semiconductors, Chinese Academy of Sciences, P.O. Box 912, Beijing 100083, China; 2 Department of Physics and Key Laboratory for Low-Dimensional Structures and Quantum Manipulation (Ministry of Education), Hunan Normal University, Changsha 410081, China; 3 Department of Physics, Zhejiang Normal University, Jinhua 321004, China; 4 Department of Physics and Electronic Science, Changsha University of Science and Technology, Changsha 410004, China

## Abstract

We investigate theoretically the Landau levels (LLs) and magneto-transport properties of phosphorene under a perpendicular magnetic field within the framework of the effective ***k***·***p*** Hamiltonian and tight-binding (TB) model. At low field regime, we find that the LLs linearly depend both on the LL index *n* and magnetic field *B*, which is similar with that of conventional semiconductor two-dimensional electron gas. The Landau splittings of conduction and valence band are different and the wavefunctions corresponding to the LLs are strongly anisotropic due to the different anisotropic effective masses. An analytical expression for the LLs in low energy regime is obtained via solving the decoupled Hamiltonian, which agrees well with the numerical calculations. At high magnetic regime, a self-similar Hofstadter butterfly (HB) spectrum is obtained by using the TB model. The HB spectrum is consistent with the LL fan calculated from the effective ***k***·***p*** theory in a wide regime of magnetic fields. We find the LLs of phosphorene nanoribbon depend strongly on the ribbon orientation due to the anisotropic hopping parameters. The Hall and the longitudinal conductances (resistances) clearly reveal the structure of LLs.

The group V element phosphorus can exist in several allotropes and black phosphorus (BP) is the most stable phase under normal conditions^1^. Recently, layered BP has attracted intensive attention because of its unique electronic properties and potential applications in nanoelectronics^2^,^3^,^4^,^5^,^6^,^7^,^8^. In the bulk form, BP is a van der Waals-bonded layered material where each layer forms a puckered surface due to *sp*^3^ hybridization^2^,^3^. BP possesses a direct band gap 0.3 eV located at Z point^3^,^4^. This direct gap increases to 1.5–2 eV when the thickness decreases from bulk to few layers and eventually monolayer via mechanical exfoliation^3^,^5^,^9^. Hence, BP is an appealing candidate for tunable photodetection from the visible to the infrared part of the spectrum^10^. Further, the field-effect-transistor (FET) based on few layer BP is found to have an on/off ratio of 10^5^ and a carrier mobility at room temperature as high as 10^3^ cm^2^/V·s^3^,^5^, which make BP a favorable material for next generation electronics.

The low energy physics of monolayer BP (phosphorene) around Γ point can be well described by an anisotropic two band ***k***·***p*** model^2^, which agrees well with a tight binding (TB) model^11^. To date, various interesting properties for phosphorene have been predicted theoretically and verified experimentally, including those related to strain induced gap modification^2^, tunable optical properties^12^, layer controlled anisotropic excitons^13^, quantum oscillations in few layers BP^14^,^15^,^16^ etc. However, the Landau levels (LLs) and magneto-transport (MT) properties of this unique anisotropic system remain unexplored.

In this work, we study the LL spectra and MT properties of phosphorene under a perpendicular magnetic field. By using an effective ***k***·***p*** Hamiltonian, we find that the LLs linearly depend both on energy index *n* and magnetic field *B* at low-field regime, which means the LLs in phosphorene are similar with that in conventional semiconductor two dimensional gases (2DEGs). Interestingly, owing to the anisotropic energy dispersions, i.e., the effective masses, the Landau splittings of conduction and valence band are different for a fixed magnetic field, and the wavefunctions corresponding to the LLs show strong anisotropic behavior. We obtain an analytical expression for the LLs in low energy regime via solving a decoupled Hamiltonian, which agrees well with the numerical data in low energy regime. At high-field regime, magneto-level spectrum, i.e., the Hofstader butterfly (HB) spectrum, is obtained by using a tight binding (TB) model. We find that the results obtained by the effective ***k***·***p*** Hamiltonian and TB model agree with each other in weak magnetic field cases. Further, we find the LLs of phosphorene nanoribbon depend strongly on the ribbon orientation due to the anisotropic hopping parameters. In order to detect those interesting magneto energy spectra, we calculate MT properties of phosphorene within the framework of the linear response theory. By using Kubo formula, we find the Hall and the longitudinal conductances (resistances) clearly reveal the structure of LLs.

## Results

### Tight binding and Low energy *k*·*p* model

**Figure 1:**
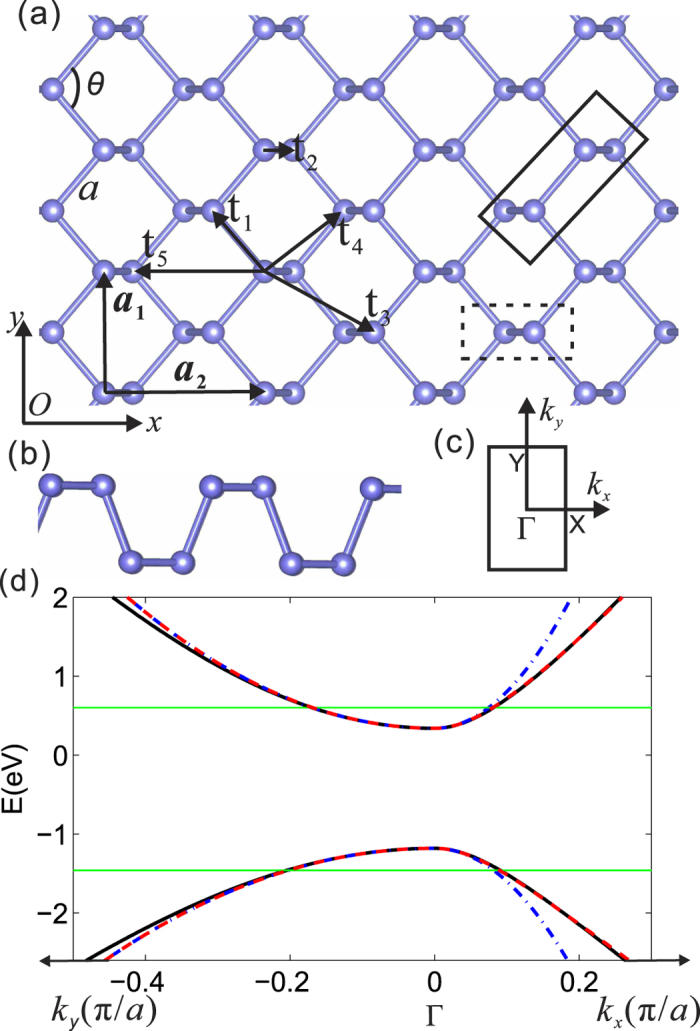
(**a**) The top view of phosphorene, *a* = 2.22 *Å* (*θ* = 96.79°) is the in plane bond length (angel), *a*_1_ (3.32 *Å*) and *a*_2_ (4.38 *Å*) the primitive vectors, *t*_*i*_ (*i* = 1, 2, 3, 4, 5) the five hopping links for TB model. (**b**) The side view of phosphorene. (**c**) The first Brillouin zone of phosphorene. (**d**) The energy dispersions of phosphorene, the black solid and red dashed lines, represent the results obtained from the TB and low energy ***k***·***p*** models, respectively. The blue dash-dotted lines represent the results obtained from the decoupled Hamiltonian (12) and the green solid line illustrates the energy regime where three Hamiltonians agree well with each other.

In the top view of phosphorene, as shown in Figure 1(a), *a*_1_ = 3.32 *Å* and *a*_2_ = 4.38 *Å* are the primitive vectors, *a* = 2.22 *Å* and *θ* = 96.79° are the in-plane bond length and bond angle^11^, respectively. The unit cell of phosphorene contains four atoms (see the solid rectangle) with two phosphorus atoms in the lower layer and the other two atoms in the upper layer. Very recently, a tight binding (TB) model of phosphorene has been proposed and is given by^11^


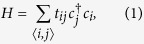


where the summation runs over all the lattice sites of phosphorene, 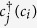 is the creation (annihilation) operator of electron on the site *j*(*i*), and *t*_*ij*_ are the hopping parameters. It has been shown that five hopping links (see Fig. 1(a)) are enough to describe the electronic band structure of phosphorene^11^. The related hopping parameters are: *t*_1_ = −1.22 eV, *t*_2_ = 3.665 eV, *t*_3_ = −0.205 eV, *t*_4_ = −0.105 eV, and *t*_5_ = −0.055 eV.

Generally, the energy dispersion of phosphorene should be described by a four band model^11^,^17^ in the TB framework. However, it can be also expressed by a two-band model due to the *D*_2*h*_ point group invariance^17^. In the two-band model, the unit cell contains two phosphorus atoms (see the dashed rectangle in Fig. 1(a)), where one is in the upper layer and the other in the lower layer. Expanding the TB model around Γ point with a coordinate rotation^17^ (*τ*_*y*_ → *τ*_*x*_, *τ*_*x*_ → *τ*_*z*_), one obtains the low energy ***k***·***p*** model for phosphorene, which reads


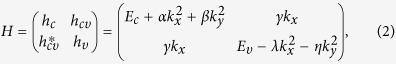


where *E*_*c*_ = 0.34 eV (*E*_*υ*_ = −1.18 eV) is the conduction (valence) band edge, *γ* = −5.2305 eV·*Å* describes the interband coupling between the conduction and valence band, parameters *α*, *β*, λ, *η* are related to the effective masses with *α* = *ħ*^2^/2*m*_*cx*_, *β* = *ħ*^2^/2*m*_*cy*_, λ = *ħ*^2^/2*m*_*υx*_, *η* = *ħ*^2^/*m*_*υy*_. Here *m*_*cx*_ = 0.793*m*_*e*_, *m*_*cy*_ = 0.848*m*_*e*_, *m*_*υx*_ = 1.363*m*_*e*_, *m*_*υy*_ = 1.142*m*_*e*_, and *m*_*e*_ is the free electron mass. The eigenvalue of this Hamiltonian is


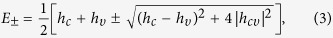


where +/− is for conduction/valence band, respectively. Similar with other low energy ***k***·***p*** models^2^,^12^, the dispersion described by Eq. (3) is strongly anisotropic. The energy gap *E*_*g*_ is *E*_*c*_−*E*_*υ*_ = 1.52 eV, which is consistent with the first principle calculations (an intrinsic energy gap around 2 to 2.2 eV minus the excition binding energy)^13^ and the recently measured optical gap^5^ 1.45 eV. Figure 1 presents the dispersion of TB (the black solid line) and the ***k***·***p*** models (the red dashed line), from which we find they agree well with each other in a quite wide energy regime. It seems the energy dispersion is linear along Γ−*X* direction (see Fig. 1(c)). However, it is actually parabolic. In the long wave limit, we have 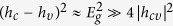. Hence, we can expand Eq. (3) around Γ point and obtain the energy dispersion of conduction and valence band, which reads


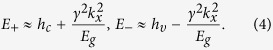


From Eq. (4), one can easily find that the dispersion near Γ point is quadratic. Owing to the interband coupling, the effective masses around Γ point along *k*_*x*_ direction are modified as 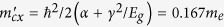, 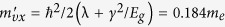. However, the effective masses around Γ point along *k*_*y*_ remain unchanged with *m*_*cy*_ = 0.848*m*_*e*_, *m*_*υy*_ = 1.142*m*_*e*_.

### Landau levels in monolayer phosphorene

When a perpendicular magnetic filed ***B*** = (0, 0, *B*) is applied, taking the Landau gauge ***A*** = (−*By*, 0, 0), we define the creation and annihilation operators as


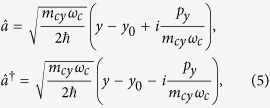


where 
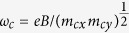
 is the frequency, 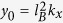 is the cyclotron center, and 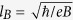 is the magnetic length. One finds Hamiltonian (2) turns to

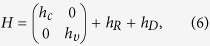


with


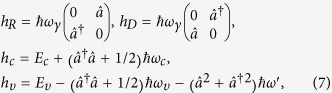


where 
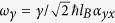
, *ω*_*υ*_ = (*r*_*x*_+*r*_*y*_)*ω*_*c*_, *ω*′ = (*r*_*x*_−*r*_*y*_)*ω*_*c*_/2, with 
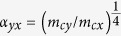
, *r*_*x*_ = *m*_*cx*_/2*m*_*υx*_ and *r*_*y*_ = *m*_*cy*_/2*m*_*υy*_. Interestingly, the second (third) term in Eq. (6) looks like the Rasshba (Dressehaus) spin-orbit interaction in conventional semiconductor 2DEG^18^. In order to understand how the non-diagonal element *h*_*R*_ and *h*_*D*_ couple the Landau levels (LLs) in conduction and valence band, we firstly simplify the Hamiltonian by ignoring the third term in *h*_*υ*_ (see Eq. (7)) since it is a second-order perturbation. It will be included in numerical calculation. In this approximation, we see that the term *h*_*R*_ couples the LL 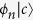 with 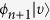, while *h*_*D*_ couples 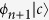 with 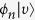, where 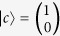, 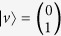, {*ϕ*_*n*_} are wave functions of the harmonic oscillator corresponding to *h*_*c*_.

When only the term *h*_*R*_ exits, we obtain 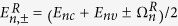, 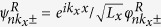 (*n* = 1, 2, …), where


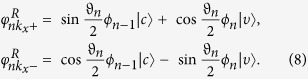


**Figure 2:**
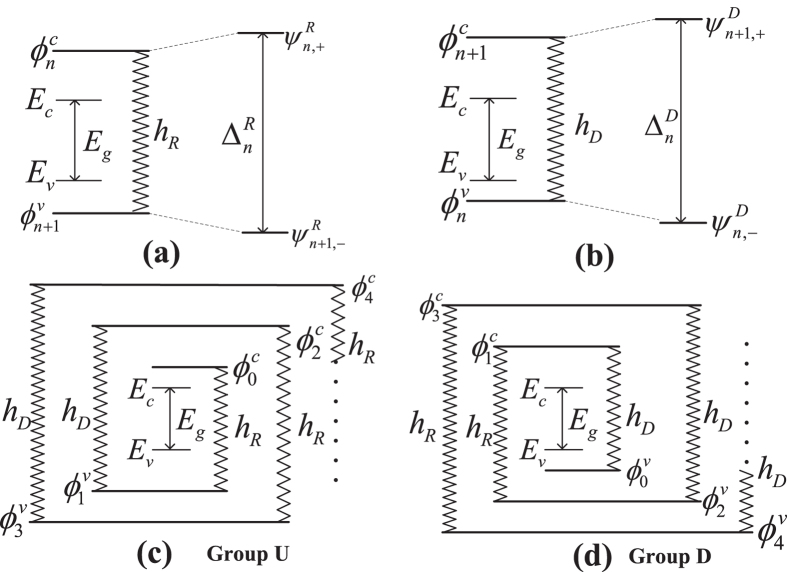
Schematic illustration of the inter-LL coupling induced by (**a**) *h*_*R*_ with 
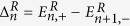
 and (**b**) *h*_*D*_ with 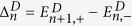; while (**c**)/(**d**) represents coupled LL in group U/D.

Here 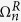 and *ϑ*_*n*_ are defined from 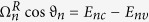, 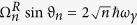. The *h*_*R*_ induces the coupling of the LL’s, which is schematically shown in Fig. 2(a). We see that both 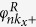 and 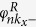 come from 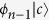 and 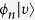. A particular eigenstate is the lowest LL in valence band 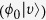, which is independent of *h*_*R*_. Meanwhile, when only *h*_*D*_ exits, we obtain 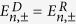, 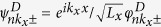 (*n* = 1, 2, …), where


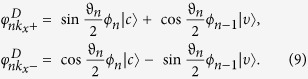


The *h*_*D*_ induces the coupling of the LL’s as schematically shown in Fig. 2(b). We see that both 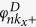 and 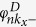 come from 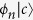 and 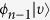. A particular eigenstate is the lowest LL in conduction band (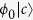), which is independent of *h*_*D*_. Therefore, when both *h*_*R*_ and *h*_*D*_ exist, the LLs are coupled into the following two groups


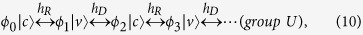


and


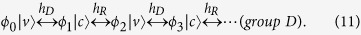


The two groups are schematically illustrated in Fig. 2(c,d). The eigenvalues and eigenvectors can be evaluated numerically by taking the eigenvectors of *h*_*c*_ in Eq. (7) as basis functions (see methods for details).

From the Hamiltonian (2), the LLs can be solved analytically in low energy regime. Although the dispersion is dominated by the off-diagonal element, we can decouple the conduction and valence band in low energy regime due to the large optical gap (1.52 eV), i.e., the weak interband coupling. The role of the off-diagonal elements can be taken into account perturbatively. The decoupled Hamiltonian reads


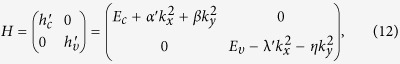


where *α*′ = *α* + *γ*^2^/*E*_*g*_, λ′ = λ + *γ*^2^/*E*_*g*_. The dispersion of this Hamiltonian is presented by the blue dash-dotted lines in Fig. 1(d). We see that in the energy regime about 300 meV (see the green solid line) with respect to the band edges the decoupled Hamiltonian agrees well with the TB and the ***k***·***p*** model. The LL of this Hamiltonian is


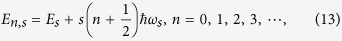


where *s* = ±1 denotes the conduction and valence band respectively, *n* represents the LL index, *E*_+/−_ = *E*_*c*/*υ*_, 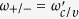 with 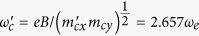 and 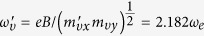, and *ω*_*e*_ = *eB*/*m*_*e*_. Our formulism for the low-energy LLs was used in the studies about the magneto-optical^19^ and transport properties^14^,^15^.

**Figure 3:**
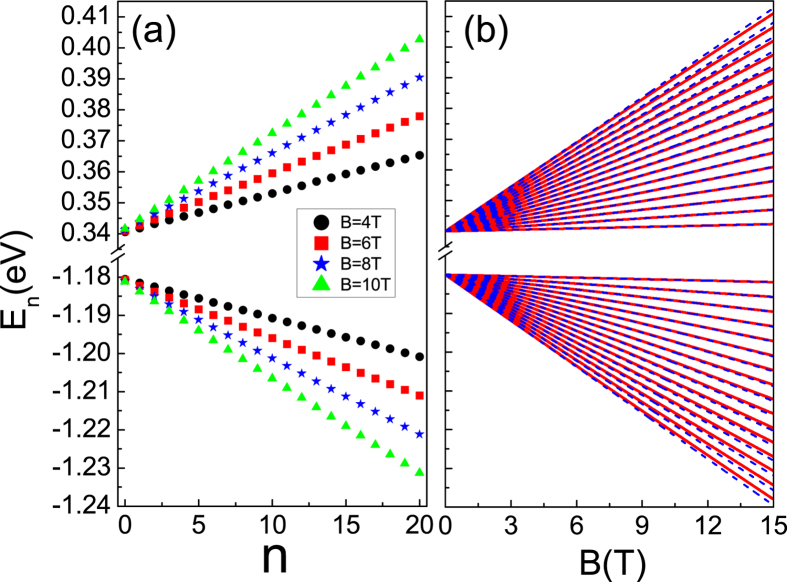
Landau levels (*E*_*n*_ in units of eV) versus (**a**) Landau energy index *n* with different magnetic field, and (**b**) magnetic field *B* for the first ten low LLs. The number of the basis function used is 200 to get convergent results. The red solid lines denote the numerical data and the blue dashed lines represent the analytical expression in Eq. (13).

Figure 3 presents the LLs versus (a) LL index *n* with different magnetic fields and (b) magnetic field *B*. The number of basis function used in the calculation is 200 to get convergent numerical results. As shown in Fig. 3(b), we find the analytical LLs (the blue dashed lines) are in good agreement with the numerical results (the red solid lines), which means the decouple Hamiltonian (12) is a good approximation in low energy regime. However, the Landau splittings of conduction and valence band are different for a fixed magnetic field (see Eq. (13)) due to the different anisotropic effective masses at zero field. Further, the Landau energies linearly depend both on LL index *n* (see Fig. 2(a)) and magnetic field *B* (see Fig. 2(b)), which is similar with that of conventional semiconductor 2DEGs since the zero field dispersion is quadratic (see Eq. (4)). Meanwhile, we find the LLs are equally spaced which can also been seen clearly in Eq. (13).

On the other hand, we find the corresponding eignevectors are anisotropic due to different effective masses along Γ−*X* and Γ−*Y* direction. In Landau gauge ***A*** = (−*By*, 0, 0), the eigenvectors are


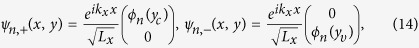


where *y*_*c*/*υ*_ = *κ*_*c*/*υ*_(*y*−*y*_0_) with 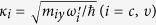. While in Landau gauge ***A*** = (0, *Bx*, 0), the eigenvectors are


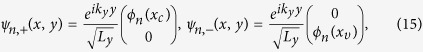


where 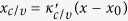, with 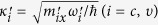. Obviously, the corresponding eigenvectors are anisotropic due to different effective masses according to Eqs. (14) and (15). Further, we will see this anisotropy more clearly in symmetry gauge. The wavefunctions in symmetry gauge are given by


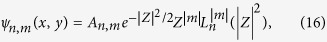


where *Z* = *X* + *iY*, 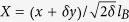, 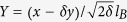, and 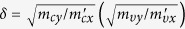 for conduction (valence) band, and 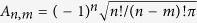 is the normalization constant, 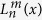 is the Laguerre polynomials.

**Figure 4:**
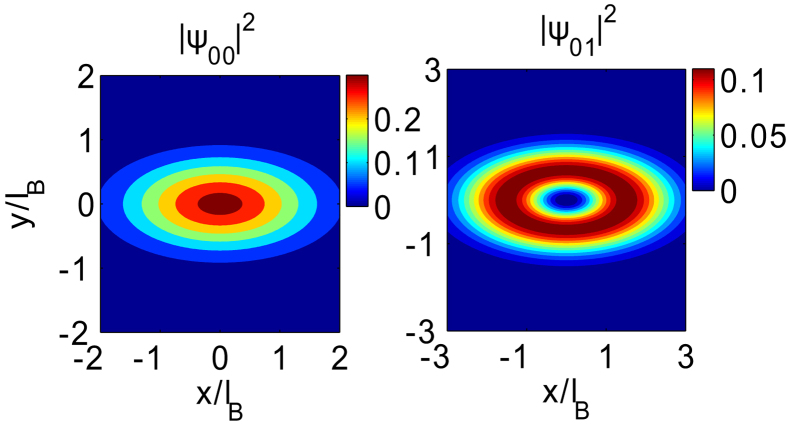
Contour plot of the spatial density distributions of the first two LLs in conduction band in symmetry gauge.

Figure 4 presents the contour plot of spatial density distributions (SDDs) corresponding to the first two LLs in conduction band. As plotted in Fig. 4, unlike the isotropic case, we find the SDDs for the first two LL are ellipses, which show strong anisotropy. The decay length of the SDDs along *x* direction is larger than that along *y* direction as the effective masses along Γ−*X* direction is smaller than that in Γ−*Y* direction (see Eq. (4)) in conduction band. The same conclusion can be drawn for SDDs corresponding to LLs in valence band.

### Hofstadter Butterfly spectrum

**Figure 5:**
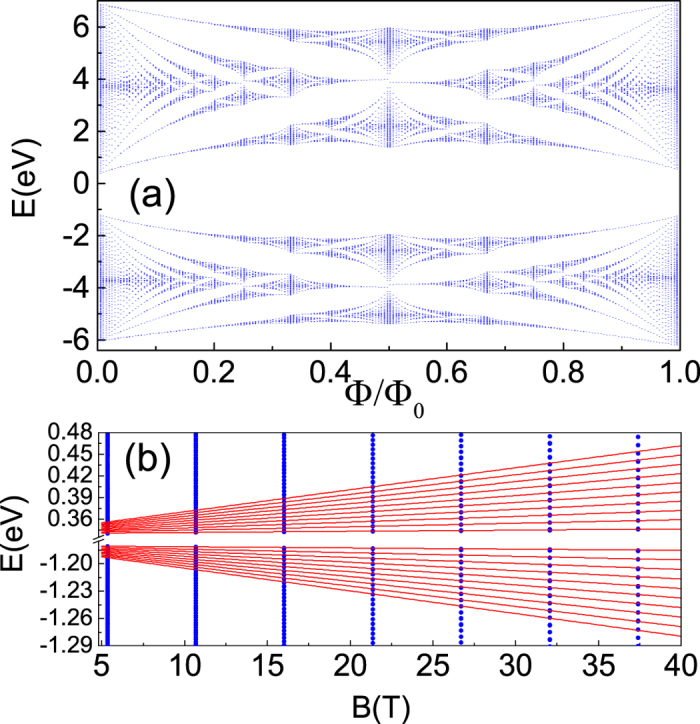
(**a**) Hofstadter butterfly (HB) spectrum of phosphorene with *q* = 199 and hopping parameters *t*_1_ = −1.22 eV, *t*_2_ = 3.665 eV, *t*_3_ = −0.205 eV, *t*_4_ = −0.105 eV and *t*_5_ = −0.055 eV. (**b**) Landau levels obtained from the TB model, i.e., the HB spectrum (the blue dots) and the ***k***·***p*** model (the red solid line) as a function a magnetic field at low field regime with *q* = 10007.

Adopting the TB model, we plot the Hofstadter Butterfly (HB) spectrum as a function of Φ/Φ_0_ (magnetic field *B*) with *q* = 199 in Fig. 5(a). As shown in Fig. 5(a), we find two gapped self-similar HB spectrum coming from the conduction and valence orbitals, respectively. Moreover, the LL energies linearly depend on magnetic field *B* at low field region, which is in line with the results obtained from the ***k***·***p*** model (see Eq. (13)). The band width of the HB spectrum in conduction and valence band is different because of the different band widths at zero field. Figure 5(b) depicts the magneto-levels i.e., the HB spectrum (the blue dots) and the LLs (the red solid lines) calculated from the ***k***·***p*** theory as a function of magnetic field at low field regime with *q* = 10007. As shown in the figure, we find they agree well with each other in wide regime of magnetic fields.

### Landau levels in phophorene nanoribbons

**Figure 6:**
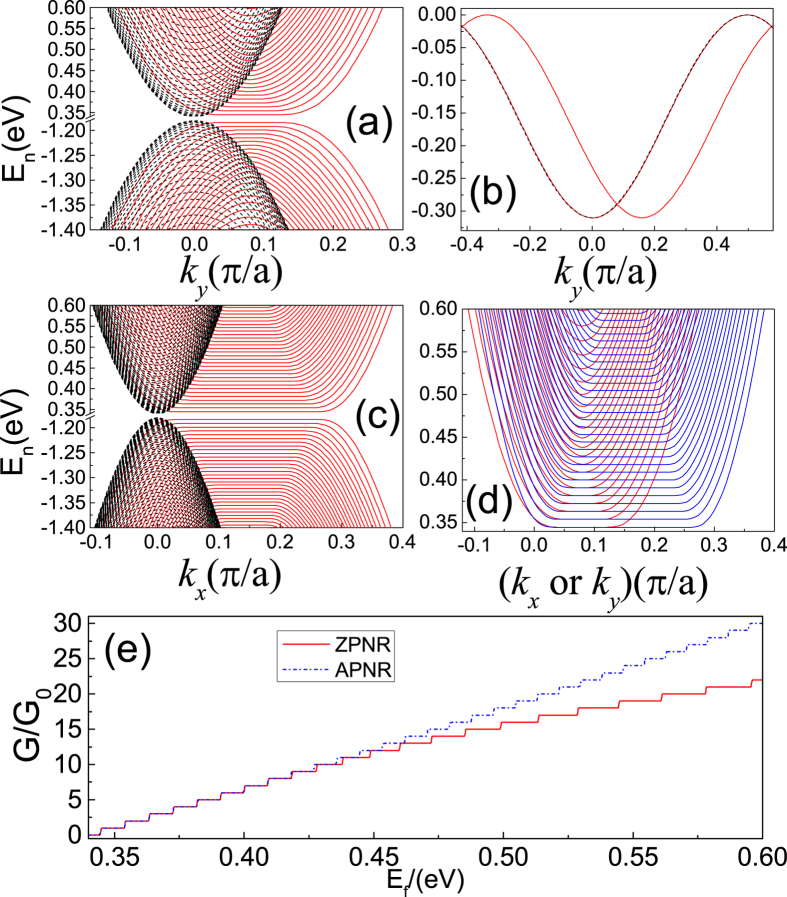
(**a**) LLs in a ZPNR with width *w* = 66.4 nm, the red solid and black dashed lines represent the energy spectra with and without external magnetic fields, respectively; *B* = 30 T (*l*_*B*_ = 4.67 nm); (**b**) The same for (a), but for topological flat band located in the bulk gap. (**c**) LLs in APNR with the same parameters used in (a). (**d**) LLs in ZPNR (red solid lines) and APNR (blue solid lines) to show clearly the anisotropic feature of the LLs; (**e**) Conductance (in unit of *G*_0_ = 2*e*^2^/*h*) as a function of Fermi energy for ZPNR (red solid line) and APNR (blue-dotted line) corresponding to (d).

Owing to the anisotropic hopping parameters in phosphorene, one can expect the edge dependent LLs in phophorene nanoribbons (PNRs). Figure 6 shows the energy spectra of a zigzag-edged PNR (ZPNR) with and without an external magnetic field. When a strong magnetic field *B* = 30 T is applied perpendicular to the ZPNR, one can clearly see the LLs. While for an armchair-edged phosphorene nanoribbon (APNR) with the same with, the LLs show different energy spacing with that in the ZPNR for the higher LLs. Comparing the energy spectra of the ZPNR and APNR, an important difference between them is that there is a topological quasi-flat band located in the bulk gap of the ZPNR^17^. There are two kinds of edges states in ZPNRs. The one is the edge states arising from the LLs in ZPNR, the other come from the topological quasi-flat band. The degeneracy of the topological qusi-flat band lifts under the influence of the magnetic field. However, since the topological quasi-flat band are mainly localized near the edges, and the decay length (~1.2 nm) is less than the magnetic length (*l*_*B*_ = 25.6 nm/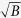 = 4.67 nm), the edge states arising from the topological quasi-flat band are almost independent of magnetic fields, i.e., no Landau quantization (see Fig. 6(b)). The LLs of PNRs depend strongly on the ribbon orientation due to the anisotropic band structure of bulk phosphorene (see Fig. 6(d)). This anisotropy of the LLs can be observed in the conductance (see Fig. 6(e)) as a function of Fermi energy (*E*_*f*_) for ZPNR (red solid line) and APNR (blue dash-dotted line).

### Magneto-transport properties of monolayer phosphorene

In order to detect the calculated magneto energy spectrum, we study the magneto-transport properties of phosphorene. In the presence of a perpendicular magnetic field, there are two contributions to magneto-conductance^20^: the Hall and collisional conductance. The former is from the non-diagonal contribution and the later from the localized states which contribute to the Shubnikov-de Haas (SdH) oscillation. In order to calculate the electrical conductance in the presence of a magnetic field, we follow the formulation of the general Liouville equation^20^. This formulation has been employed successfully in electron transport for conventional semiconductor 2DEG^18^,^20^, and more recently in graphene^21^ and MoS_2_^22^.

Within linear response theory, the Hall conductance is


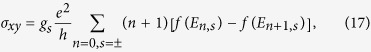


where *g*_*s*_ = 2 for the spin degree of freedom. At low temperature, the Hall conductance turns


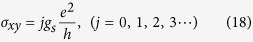


where *j* is the filling factor. This result is the same as that for a conventional semiconductor 2DEG^18^,^20^, since the zero field dispersion in low energy regime is quadratic (see Eq. (4)). To obtain the longitudinal conductance, we assume that electrons are elastically scattered by randomly distributed charged impurities since this type of scattering is dominant at low temperatures. The longitudinal conductance is


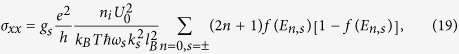


where *U*_0_ = *e*^2^/4*πε*_0_*ε*_*r*_, *n*_*i*_ is the impurity concentration, *k*_*s*_ is the screening wavevector, *ε*_*r*_ is the dielectric constant, and *ε*_0_ is the dielectric permittivity.

**Figure 7:**
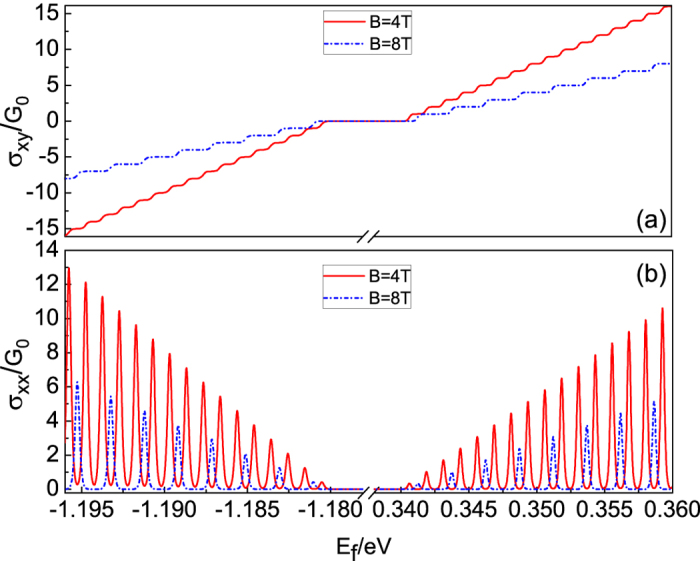
(**a**) Hall conductance *σ*_*xy*_ (in unit of *G*_0_ = 2*e*^2^/*h*) and (**b**) longitudinal conductance versus Fermi energy *E*_*f*_ (in unit of eV) with different magnetic fields. The parameters used are: temperature *T* = 1 K, impurity concentration *n*_*i*_ = 2 × 10^8^ cm^−2^, screen potential vector *k*_*s*_ = 5 × 10^7^ m^−1^, Boltzman constant *k*_*B*_ = 1.38 × 10^23^ J/K, and dielectric constant *ε*_*r*_ = 10.2.

Figure 7 shows (a) the Hall (*σ*_*xy*_) and (b) the longitudinal conductances (*σ*_*xx*_) as a function of Fermi energy (*E*_*f*_) for two different magnetic fields *B* = 4 T and 8 T, respectively. Other parameters used are temperature *T* = 1 K, impurity concentration *n*_*i*_ = 2 × 10^8^ cm^−2^, screen potential vector *k*_*s*_ = 5 × 10^7^ m^−1^, Boltzman constant *k*_*B*_ = 1.38 × 10^23^ J/K, and dielectric constant *ε*_*r*_ = 10.2^23^. As plotted in Fig. 7(a), we find that Hall conductance is strictly quantized due to the quantized LLs. It increases one by one in the unit of *G*_0_ = 2*e*^2^/*h* with the increasing of Fermi energy since the LLs are filled one by one. Therefore, we observe the integer Hall plateaus at 0, ±2, ±4, ±6, … in Hall conductance. This is similar with that in conventional semiconductor 2DEG^18^. Moreover, the Hall conductance reveals the LLs clearly since the transitions of the plateaus happen to be the energy value of LLs (see Fig. 3(a)). Further, for a fixed magnetic field, the width of the plateaus is equal since the LL spacings of two adjacent LLs are equal according to Eq. (13). As depicted in Fig. 7(b), the longitudinal conductance shows that (i) pronounced peaks appear when the Fermi energy coincides with the LLs, and (ii) a well splitting SdH oscillation can be observed, which corresponds to the LLs (see Fig. 3(a)). Meanwhile, the amplitude of longitudinal conductance increases with the increasing of the Fermi energy because of the larger scattering rate of LLs with higher index. Moreover, the intervals between the peaks are equal since the LLs are equally spaced according to Eq. (13).

**Figure 8:**
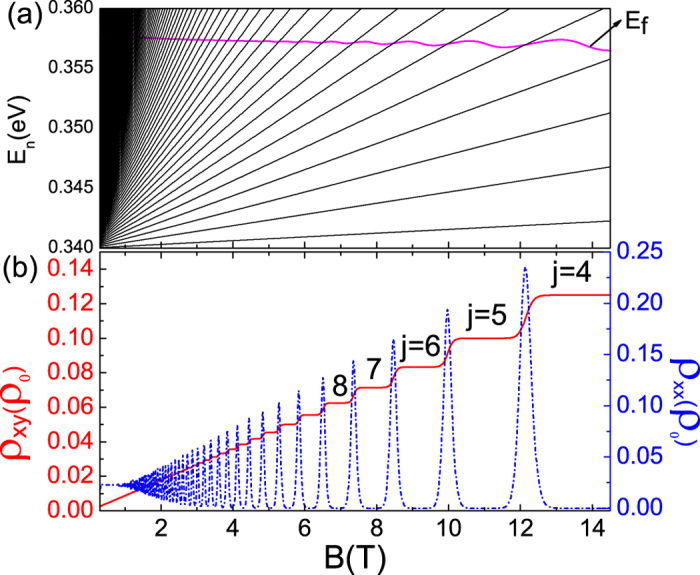
(**a**) Electron Fermi energy as a function of magnetic field for a fixed electron concentration *n*_*e*_ = 1.45 × 10^12^ *cm*^−2^, (**b**) Hall resistance (the red solid line) and magneto longitudinal resistance (the blue dash-dotted line) corresponding to (**a**), the resistance unit *ρ*_0_ is *h*/*e*^2^. The other parameters used are the same as those in Fig. 7.

Figure 8 presents the Fermi energy spectra and resistances as a function of magnetic field for a given electron concentration *n*_*e*_ = 1.45 × 10^12^ cm^−2^. Generally, the Hall (*ρ*_*xy*_) and the longitudinal resistances (*ρ*_*xx*_) can be detected directly via Hall measurement^3^,^14^. As shown in Fig. 8(b), at low magnetic field, the Hall resistance linearly depend on the magnetic field and the longitudinal one is a constant. However, at the high magnetic field regime, the Hall resistance is strictly quantized with Hall plateaus due to Landau quantization. It increases (in unit of *ρ*_0_ = *h*/*e*^2^) one by one with increasing magnetic field since the LLs leak out of the Fermi level one by one (see Fig. 8(a)). This is also reflected in the transitions of filling factor (see Fig. 8(b)). Therefore, we observe plateaus at 1/8, 1/10, 1/12, 1/14, …, in Hall resistance corresponding to filling factor *j* = 4, 5, 6, 7, …, with the decreasing of magnetic field. Meanwhile, we find a clear SdH oscillation in longitudinal resistance. The amplitude of longitudinal resistance increases with the magnetic field since it is proportional to *B*^2^. However, this oscillation is quenched in low magnetic field due to tiny LL splittings in weak field cases.

## Discussion

Our results about the LL spectrum in phosphorene can be also applied to multilayer BPs since the ***k***·***p*** Hamiltonians for the multilayer ones are similar with that for phosphorene. A very recent paper^24^ demonstrates that the low energy LLs in bulk phosphorus also depend linearly both on the LL index *n* and magnetic field *B*. Meanwhile, this result has been verified in several recent magneto transport experiments^14^,^15^,^16^. Our results have been employed to illustrate the absence of non-trivial Berry’s phase of LLs in multilayer BPs^14^.

In summary, we studied theoretically the Landau levels and magneto-transport properties of phosphorene under a perpendicular magnetic field within the framework of an effective ***k***·***p*** Hamiltonian and TB model. In the low field regime, we found that the LLs linearly depend both on the LL index *n* and magnetic field *B*, which is similar with that of conventional semiconductor two-dimensional electron gas. For a fixed magnetic field, the Landau splittings of conduction and valence band are different and the wavefunctions corresponding to the LLs show strong anisotropic behavior due to the anisotropic effective masses. We obtained an analytical expression for the LLs in low energy regime via solving a decoupled Hamiltonian. This analytical solution agrees well with the numerical results. At high magnetic regime, a self-similar Hofstadter butterfly (HB) spectrum was obtained by using the TB model. The HB spectrum is in good agreement with the LLs calculated from the effective ***k***·***p*** theory in a wide regime of magnetic fields.

Further, we found the LLs of phosphorene nanoribbons (PNRs) depend strongly on the ribbon orientation due to the anisotropic hopping parameters. There are two kinds of edge states in ZPNRs under a perpendicular magnetic field. The one is the edge states arising from the LLs, the other comes from the topological flat band. The second edge states are almost independent of magnetic fields because their decaying length is less than the magnetic length *l*_*B*_. Moreover, the Hall and the longitudinal conductances (resistances) clearly reveal the structure of LLs in phosphorene sheet.

## Methods

### Basis function expansion

The eigenvalues and eigenvectors can be evaluated numerically by taking the eigenvectors of *h*_*c*_ in Eq. (7) as basis functions. In this basis, the wavefunction of the system can be expressed as


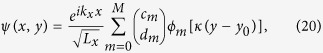


where 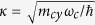, 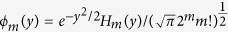 is the harmonic oscillator wave functions. Then, we can diagonalize the Hamiltonian numerically in a truncated Hilbert space and obtain the eigenvalues as well as the eigenvectors.

### HB spectrum and LLs in PNRs

In the TB framwork, when the phosphorene sample subjected to a perpendicular magnetic field, a Peierls phase should be added to the hopping parameter, which reads


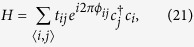


where 
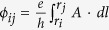
 is the Peierls phase. It was first shown by Hofstadter^25^ that the energy spectrum in this case depends on a rational dimensionless parameter *p*/*q*, where *q* is a prime number and *p* runs from 1 to *q*. This dimensionless parameter is the ratio of magnetic flux through one unit cell (Φ = *BS*) to the magnetic flux quantum (Φ_0_ = *h*/*e* = 4.14 × 10^−15^ T m^2^), where *S* is the area of a unit cell. The energies plotted as a function of Φ/Φ_0_ form a beautiful Hofstadter Butterfly (HB) spectrum. By using Eq. (21), one will arrive the the Haper’s equation and find it is periodic in 2*q*^26^. The HB spectrum is obtained numerically by getting the eigenvalues of a matrix with dimension of 4*q* × 4*q* at each ***k*** point in the magnetic Brillouin zone. A sufficiently large *q* is needed if one wants to compare this HB spectrum with the results obtained from the low energy ***k***·***p*** model due to the large magnetic flux quanta. Further, one can obtain the LLs in PNRs by applying proper boundary conditions to Eq. (21).

### Hall and longitudinal conductance (resistance)

Within linear response theory, the Hall conductance in Kubo-Greenwood formula reads^20^


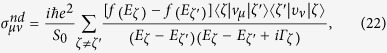


where *μ*, *v* = *x*, *y*, *S*_0_ = *L*_*x*_*L*_*y*_ is the phosphorene sample area, with the size *L*_*x*_ (*L*_*y*_) in *x*(*y*)-direction, 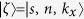 the single electron state in Eq. (14) as we are interested in the low energy transport, 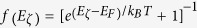 the Fermi-Dirac distribution function with Boltzman constant *k*_*B*_ and temperature *T*, 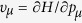 the component of group velocity. The sum runs over all states 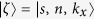 and 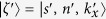 with *ζ* ≠ *ζ*′. The infinitesimal quantity Γ_*ζ*_ accounts for the finite broadening of the LLs, which is assumed approximately the same for all states^21^. In our work, we take Γ_*ζ*_ = 0 in order to obtain a transparent result for Hall conductance. With the help of Eq. (14), one can obtain Eq. (17) easily.

To obtain the longitudinal conductance, we assume that electrons are elastically scattered by randomly distributed charged impurities, as this type of scattering is dominant at low temperatures. The longitudinal conductance in Kubo-Greenwood formula is given by^20^,^21^


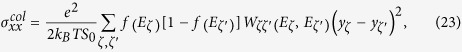


where *W*_*ζζ*′_ is the scattering rate between single-electron states 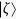 and 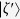. Conduction occurs via transitions through spatially separated states from *y*_*ζ*_ to *y*_*ζ*′_, where 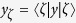 is the expectation value of *y* coordinate. This means that the longitudinal conductance arises from the migration of the cyclotron orbit because of scattering by charged impurities. The scattering rate *W*_*ζζ*′_ is


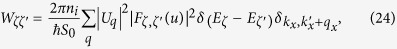


where 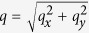, 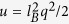, *n*_*i*_ is the impurity density, 
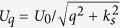
 the Fourier transform of the screened impurity potential 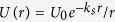 with *U*_0_ = *e*^2^/4*πε*_0_*ε*_*r*_, *k*_*s*_ is the screening wavevector, *ε*_*r*_ is the dielectric constant, and *ε*_0_ is the dielectric permittivity. Furthermore, if the impurity potential is strongly short ranged (of the Dirac *δ*-type function), one may use the approximation 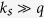 and *U*_*q*_ ≈ *U*_0_/*k*_*s*_. As the collision is elastic and the eigenvalue is independent on *k*_*x*_, only the transitions *n*→*n* are allowed. Then, one can obtain Eq. (19) directly by using Eq. (14).

Moreover, one can obtain the Hall resistance and the longitudinal one with the conductances (*σ*_*xy*_ and *σ*_*xx*_) via expressions of *ρ*_*xy*_ = *σ*_*xy*_/*S* and *ρ*_*xx*_ = *σ*_*xx*_/*S*, where 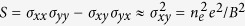 18,20,21, and *n*_*e*_ is the electron concentration. For a fixed Fermi energy (*E*_*f*_), *n*_*e*_ is given by 
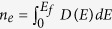
, where 
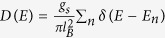
 is the density of states. Note, no matter which wavefunction (Eq. (14) or (15)) is used in the calculation, one will obtain the same results as the conductances are gauge independent.

## Additional Information

**How to cite this article**: Zhou, X. Y. *et al.* Landau levels and magneto-transport property of monolayer phosphorene. *Sci. Rep.* **5**, 12295; doi: 10.1038/srep12295 (2015).

## References

[b1] Nishii T. *et al.* Synthesis and characterzation of black phosphorus intercaltion compounds. Synth. Met. 18, 559–564 (1987).

[b2] Rodin A. S. *et al.* Strain-Induced Gap Modification in Black Phosphorus. Phys. Rev. Lett. 112, 176801 (2014). 24836264 10.1103/PhysRevLett.112.176801

[b3] Li Likai *et al.* Black phosphorus field-effect transistors. Nat. Nanotech. 9, 372–377 (2014). 10.1038/nnano.2014.35 24584274

[b4] Han C. Q. *et al.* Electronic structure of black phosphorus studied by angle-resolved photoemission spectroscopy. Phys. Rev. B 90, 085101 (2014).

[b5] Liu Han *et al.* Phosphorene: An Unexplored 2D Semiconductor with a High Hole Mobility. Acs Nano. 8, 4033–4041 (2014). 24655084 10.1021/nn501226z

[b6] Churchill H. O. *et al.* Two dimensional crystals phosphorus joins the family. Nat. Nanotech. 9, 330 (2014). 10.1038/nnano.2014.85 24801536

[b7] Reich E. S. Phosphorene excites materials scientists. Nature 506, 19 (2014). 24499900 10.1038/506019a

[b8] Castellanos-Gomez Andres *et al.* Isolation and characterization of few-layer black phosphorus. 2D Mater. 1, 025001 (2014).

[b9] Lu Wanglin *et al.* Plasma-assisted fabrication of monolayer phosphorene and its Raman characterization. Nano. Res. 7, 853–859 (2014).

[b10] Buscema M. *et al.* Fast & Broadband Photoresponse of Few-Layer Black Phosphorus Field-Effect Transistors. Nano. Lett. 14, 3347–3352 (2014). 24821381 10.1021/nl5008085

[b11] Rudenko A. N. & Katsnelson M. I. Quasiparticle band structure and tight-binding model for single- and bilayer black phosphorus. Phys. Rev. B 89, 201408(R) (2014).

[b12] Low T. *et al.* Tunable optical properties of multilayer black phosphorus thin films. Phys. Rev. B 90, 075434 (2014).

[b13] Tran Vy *et al.* Layer-controlled band gap and anisotropic excitons in few-layer black phosphorus. Phys. Rev. B 89, 235319 (2014).

[b14] Li Likai *et al.* Quantum Oscillations in Black Phosphorus Two-dimensional Electron Gas. *arXiv*: 1411.6572 (2014).

[b15] Chen Xiaolong *et al.* High quality sandwiched black phosphorus heterostructure and its quantum oscillations. *arXiv*: 1412.1357 (2014). 10.1038/ncomms8315 PMC4557360 26099721

[b16] Tayari V. *et al.* Two-Dimensional Magnetotransport in a Black Phosphorus Naked Quantum Well. *arXiv*: 1412.0259 (2014). 10.1038/ncomms8702 PMC4506510 26151889

[b17] Ezawa M. Topological origin of quasi-flat edge band in phosphorene. New. J. Phys. 16, 115004 (2014).

[b18] Yang W. & Chang K. Magnetotransport in two-dimensional electron gas: The interplay between spin-orbit interaction and Zeeman splitting. Phys. Rev. B 73, 045303 (2006).

[b19] Yuan Shenjun *et al.* Transport and optical properties of single- and bilayer black phosphorus with defects. Phys. Rev. B 91, 115436 (2015).

[b20] Ando Tsuneya & Uemura Yasutada Theory of quantum transport in a two-dimensional electron system under magnetic fields. J. Phys. Soc. Jpn. 36, 959 (1974).

[b21] Krstajic P. M. *et al.* Integer quantum Hall effect in gapped single-layer graphene. Phys. Rev. B 86, 115432 (2012).

[b22] Zhou X. Y. *et al.* Magnetic control of valley and spin degrees of freedom via magnetotransport in n-type monolayer MoS_2_. Journal of Physics: Condensed Matter 26, 485008 (2014). 25373934 10.1088/0953-8984/26/48/485008

[b23] Nagahama T. *et al.* Optical Determination of Dielectric Constant in Black Phosphorus. J. Phys. Soc. Jpn. 54, 2096 (1985).

[b24] Fei Ruixiang *et al.* Topological Protected Dirac Cones in Compressed Bulk Blakc Phosphorus. *arXiv*: 1501.00706 (2015).

[b25] Hofstader Douglas R. Energy levels and wave functions of Bloch electrons in rational and irrational magnetic fields. Phys. Rev. B 14, 2339 (1976).

[b26] Gumbs G. & Fekete P. Hofstadter butterfly for the hexagonal lattice. Phys. Rev. B 56, 3787 (1997).

